# Incorporation of the New *anti*-Octadecaborane Laser Dyes into Thin Polymer Films: A Temperature-Dependent Photoluminescence and Infrared Spectroscopy Study

**DOI:** 10.3390/ijms23158832

**Published:** 2022-08-08

**Authors:** Tereza Capkova, Barbora Hanulikova, Jakub Sevcik, Pavel Urbanek, Jan Antos, Michal Urbanek, Ivo Kuritka

**Affiliations:** Centre of Polymer Systems, Tomas Bata University in Zlin, Tr. Tomase Bati 5678, 760 01 Zlín, Czech Republic

**Keywords:** infrared reflection–absorption spectroscopy, photoluminescence spectroscopy, borane cluster, thin film, transition temperature

## Abstract

New *anti*-octadecaborane(22) laser dyes have been recently introduced. However, their application in solid thin films is limited, despite being very desirable for electronics. Spectroscopic methods, photoluminescence (PL), and infrared reflection–absorption spectroscopy (IRRAS), are here used to reveal structural responses to a temperature change in thin polymer films made of π- and σ-conjugated and non-conjugated polymers and *anti*-octadecaborane(22) and its tetra-alkylatedderivatives. It has been observed that borane clusters are not firmly fixed within polymer matrices and that their ability for diffusion out of the polymer film is unprecedented, especially at higher temperatures. This ability is related to thermodynamic transitions of polymer macromolecular chains. PL and IRRAS spectra have revealed a clear correlation with β-transition and α-transition of polymers. The influence of structure and molecular weight of a polymer and the concentration and the substitution type of clusters on mobility of borane clusters within the polymer matrix is demonstrated. A solution is proposed that led to an improvement of the temperature stability of films by 45 °C. The well-known spectroscopic methods have proved to be powerful tools for a non-routine description of the temperature behavior of both borane clusters and polymer matrices.

## 1. Introduction

The photoluminescence (PL) behavior of thin films made of π- or σ-conjugated polymers is crucial for their use in optoelectronic devices, such as LEDs. Moreover, if the PL is evoked or supported with a dopant (low molecular compound), it is essential to attain a firm attachment of the dopant in the polymer matrix. Over the years, dopant molecules have become an important part of thin active layers because they can improve charge balance [1], serve as co-emitter, triplet luminophore [2,3] or weak molecular dopant [4]. The dopant is often added into film-forming solutions, and its incorporation in a polymer matrix is achieved physically. The stability of the dopant–matrix system, i.e., low diffusion rate of a dopant, is dependent on intermolecular interactions, the molecular weight of the polymer, and polymer properties (such as flexibility of chains, the bulkiness of side groups, transition temperatures, etc.). Recently, a new class of *anti*-octadecaborane laser dyes has been introduced that offers the best potential yet for application in electronics due to their high quantum yield (close to 1) and extreme stability when used as laser dyes [5]. However, reports on their application have become delayed, as several serious obstacles have been experienced during the preparation of LEDs using these materials [6].

Due to dopant fixation in the film matrix at higher temperatures, thermodynamic transitions of the polymer must be taken into account. Thin polymer films manifest different behavior when exposed to increased temperatures compared to bulk systems due to their spatial confinement [7]. It has already been discovered that films with thickness below 100 nm show substantial deviation of their glass transition temperature (*T_g_*) [8], which is dependent on 1) the type of film, either free-standing [9,10] or substrate-supported [11], (2) polymer–substrate interactions that can increase the *T_g_* of a film above a bulk value and broaden its range [12], or (3) the molecular weight of the polymer [13]. However, the point of glass transition must be grasped in more detail. It is a known fact that glass transition is not represented with a single temperature value, and the entire glass transition domain cannot be characterized with a single temperature range either. Localized movements along macromolecular chains are of lower importance in bulk polymer systems, and glass transition is described by a release of chain segment movements. However, thin polymer films with nano-scale thickness and spatial constraints are influenced by the localized movements of side groups and even by vibrational movements of atomic groups. The glass transition domain is thus divided into several dynamic sub-domains, which describe (1) α-transition, characterized with *T_α_* and usually considered as *T_g_* (also of bulk polymer) connected with a release of segmental movements, which are cooperative [14,15]; (2) β-transition, characterized with *T_β_* related with localized movements of side groups as enabled by the structure of a polymer (e.g., rotation of single bonds) [16]; (3) γ-transition characterized with *T_γ_*, which reflects non-cooperative vibrational movements of atoms (i.e., stretching and bending) [17,18]. Their values are correspondingly sorted *T_α_ > T_β_ > T_γ_*, and *T_α_* is usually close to bulk *T_g_*. From this point of view, a spectroscopic characterization is a suitable way to describe thin films. In particular, molecular spectroscopies such as absorption and PL spectroscopy are sensitive to α- and β-relaxation, as they can follow the changes in structure via electronic interactions and changes in the length of conjugated chain segments [19] or infrared spectroscopy in reflection–absorption mode, which is directly connected with dipole moment changes and vibrational movements [20]. Spectroscopic ellipsometry has been used for a glass transition description using a film thickness and a linear thermal expansion coefficient [21,22]. Even mechanical spectroscopy has proved to be a valuable tool for detecting the glass transition of thin films [23]. The results of the spectroscopic investigation have corresponded throughout various studies and therefore have shown their reliability.

The purpose of this study is to describe *anti*-octadecaborane(22) (in-text abbrev., B) and its derivatives 3,3′,4,4′-Me_4_-*anti*-B_18_H_18_ (BMe) and 3,3′,4,4′-Et_4_-*anti*-B_18_H_18_ (BEt) in thin polymer films and their response to a change in the film temperature. These borane clusters were chosen to represent the new borane-based laser dyes because they have shown promising results of stable PL with efficient blue emission [24,25]. However, our first observation within photoluminescence spectra indicated that the intensity of emission maximum of borane clusters decreased as the temperature of the film increased until it fell below the detection limit at a specific temperature. This temperature correlates with glass transition domains of used PL polymers. The drop of the borane cluster PL intensity is probably connected with accelerated diffusion within the film when macromolecular chains rearrange through β- and α-relaxation. Considering that we have already found the polarization-modulation infrared reflection–absorption spectroscopy (PM-IRRAS) method to be a suitable way to describe sub-*T_g_* transitions in temperature domains lower than *T_α_* of polystyrene [26], we applied this method to borane cluster instability in polymer thin films when heated. Although the diffusion and/or migration of low molecular species within the polymer matrix of a thin film has been described thoroughly [27,28], the extent and the rate of borane cluster migration out of our studied films was unprecedented. Such volatility of a molecular additive is detrimental to any potential application in thin film electronics. For this reason, we investigated the roles of polymer structure and molecular weight, concentration of borane clusters, and type of borane cluster substitution in diffusion of borane clusters within the film. The results correlated with β- and α-transition of polymers. The study involved thin films made of π- and σ-conjugated polymer and a non-luminescent polymer (polystyrene), whose behavior under heating perfectly matches with proposed relations between segmental and side-chain movement and molecular vibrations and diffusion of borane clusters. We also propose a solution that improves a stability of borane clusters in thin films and that does not influence a chemical structure in clusters that would lead to change in their unique PL properties. The solution involves an adjustment of thin film design via a preparation of a thin film (with dopant) overlaid with another polymer.

## 2. Results and Discussion

**Figure 1:**
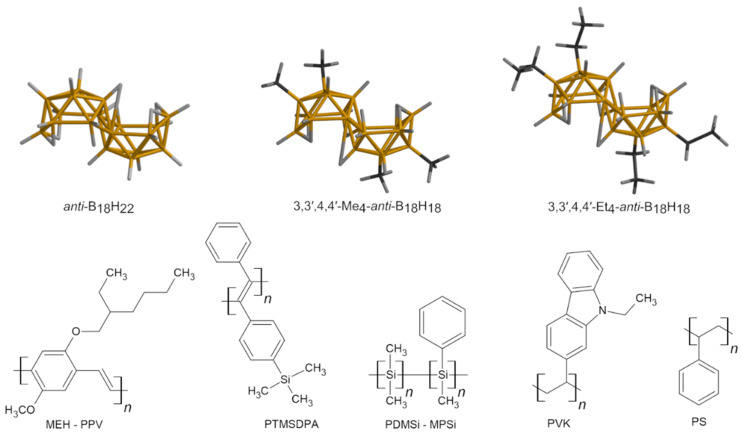
Structures of borane clusters (boron, orange; carbon, black) and structural formulas of polymers.

The problematics of thin films prepared from polymer and low molecular species, here represented with luminescent polymers and PS with borane clusters and their behavior at an increased temperature, are affected by three main factors: (1) polymer properties that characterize glass transition temperature domain (molecular weight, rigidity/flexibility of polymer chains, free volume of the system); (2) borane cluster (type of substitution –*anti*-B_18_H_22_ or its tetraethyl- or tetramethyl- derivative, concentration of the borane cluster in the film); (3) thin film properties (thickness, type of a substrate). The given film properties are not considered as factors that should substantially influence the results of the spectroscopic measurement because the film thickness is higher than 100 nm (except two films for PL measurement, where thickness is 46 nm and 60 nm) and no strong polymer–substrate interactions are expected due to the chemical character of the substrates. On the other hand, chemical nature of borane clusters and polymers is regarded as a key factor determining spectroscopic response of thin films. Structures of all clusters and polymer structural formulas are depicted in Figure 1.

### 2.1. PL Spectroscopy Experiments

PL spectroscopy is an essential method for the analysis of thin films consisting of PL polymer and *anti*-B_18_H_22_ because this centrosymmetric isomer of octadecaborane(22) manifests a blue colored emission band. In detail, the *anti*-B_18_H_22_ spectral band has been detected at 407 nm with quantum yield (QY) 0.97 when measured in hexane [24]. Moreover, this emission maximum (*λ_maxEm_*) is tunable by adjustment of *anti*-B_18_H_22_ molecular structure via substitution of hydrogens with e.g., alkyl- groups 3,3′,4,4′-Me_4_-*anti*-B_18_H_18_ and 3,3′,4,4′-Et_4_-*anti*-B_18_H_18_ (*λ_maxEm_* = 424 and 427 nm, respectively, QY = 1, both) [25], sulphur 4,4′-(HS)_2_-*anti*-B_18_H_20_ (*λ_maxEm_* = 536 nm) [29], or iodine 4,4′-I_2_-*anti*-B_18_H_20_ (λ_maxEm_ = 545 nm with QY = 0.71) [30].

However, *anti*-B_18_H_22_ also carries downsides that are mainly connected with low quantum yields of PL in π-conjugated systems of both solvents and thin polymer films, such as PS film with *anti*-B_18_H_22_ prepared from toluene solution. The reason for the poor PL performance has been found in bridging hydrogens of *anti*-B18H22, which can create π-stacking interactions within the system polymer–borane cluster or solvent–borane cluster. A way of eliminating this unwanted behavior in π-conjugated systems is a chemical adjustment of *anti*-B18H22 by replacing hydrogens with larger substituents, such as methyl- or ethyl-, as demonstrated in our previous work [6].

**Figure 2:**
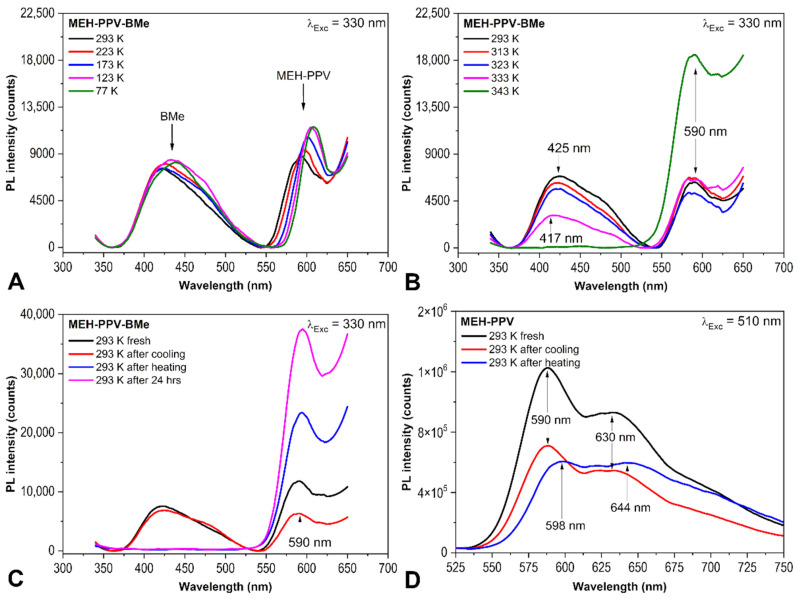
Emission spectra of MEH-PPV+BMe thin film (**A**–**C**) and MEH-PPV thin film (**D**). Cooling of the film (**A**), heating of the film (**B**), comparison of spectra after thermal exposure and after next 24 h (**C**).

In the present PL investigation, BMe (3,3′,4,4′-Me_4_-*anti*-B_18_H_18_) was examined within the thin film MEH-PPV+BMe with a thickness of 60 ± 1 nm and the weight ratio of MEH-PPV:BMe equal 2:1. Attention was paid to the behavior of emission spectra of MEH-PPV+BMe at lower/higher temperatures than RT to reveal effects connected with the thermodynamic transitions of MEH-PPV. Regarding the fact that α-transition of MEH-PPV has been reported at 340 K (67 °C), while β- transition connected with movements of side groups (predominantly of -CH_2_-) at 200–220 K (−73 °C to −53 °C) [31], the PL spectra were measured in the temperature range 77–343 K (−196 °C to 70 °C). Figure 2 shows emission spectra of the MEH-PPV+BMe and MEH-PPV films, which were collected with excitation wavelengths 330 nm and 510 nm, respectively. Firstly, MEH-PPV+BMe thin film was analyzed at decreasing temperatures, given in Figure 2A. As can be seen, two emission bands, which belong to BMe and MEH-PPV, are distinguished at 425 nm and 590 nm (20 °C), respectively. There is a clear redshift from 590 nm to 610 nm and a subsequent PL intensity increase for MEH-PPV that agrees with β-transition of the polymer as proved with the most pronounced shift between 223 K (−50 °C) and 173 K (−100 °C), corresponding to a change in PL intensity by 1307 counts. The emission band of BMe does not react at the lower temperature with so significant PL intensity change. However, a shift of the BMe emission maximum from 420 nm to 440 nm is also observed. Secondly, the emission spectra of the MEH-PPV+BMe film measured at 293 K (20 °C) and 343 K (70 °C) are substantially different, as given in Figure 2B. The emission band of MEH-PPV does not shift, and its maximum is located at 590 nm throughout the heating. Moreover, the PL intensity of this band is approximately doubled at 343 K. This observation can be identified with α-transition of this polymer that occurs between 333 K and 343 K. As depicted in Figure 2C,D, the change in PL intensity of MEH-PPV spectral band is either positive (film with BMe, with excitation wavelength 330 nm) or negative (film without BMe, with excitation wavelength 510 nm); this is probably connected with planarization of MEH-PPV chain segments in the thin film, which has been observed even below *T_α_* [32] and with an increase in intramolecular interactions [33]. Another significant change, which occurred during heating, was found at 425 nm, assigned to the BMe emission band. Surprisingly, the PL intensity of the BMe band rapidly decreased to zero between 323 K (50 °C) and 343 K (70 °C), and a minor blue shift was detected before BMe spectral band disappeared utterly. A broadening of the energy gap could be correlated with temperature-dependent electron lattice interactions [34]. These results manifest the instability of the borane cluster in the thin film at a higher temperature, especially at a temperature higher than *T_α_* of MEH-PPV. Since the α-transition is connected with an increase in the polymer-free volume and release of macromolecular chain segments from the frozen state, BMe, as a small molecule, might have been released from the grip of MEH-PPV chains, which triggered the diffusion of BMe within the film to its surface and out of the film. On the other hand, MEH-PPV gained a higher emission intensity at 590 nm within a heating procedure, which further increased after the next 24 h at RT. This PL increase is presumably encouraged by BMe elimination because it serves as an absorber. Without borane clusters in the polymer matrix that absorb preferably, more photons can be used for MEH-PPV excitation. Compared with MEH-PPV film without BMe, the course of emission spectra diverges after the heating procedure, proving the effect of BMe elimination. Moreover, the decline of BMe intensity and its inability to restore its initial PL intensity after either returning to 293 K or 24-h long conditioning at 293 K indicates that the borane cluster did diffuse out of the thin film. Similar behavior was observed with the excitation spectra given in Supplementary Materials (Figure S1). The diffusion of borane cluster out of the film was also confirmed visually to verify that this effect involves the entire film. The prepared thin film MEH-PPV+BMe was exposed to UV radiation under UV lamp and a clear blue emission was observed. After heating, the blue color of films was not observable anymore, as evidenced by photographs in Supplementary Materials in Figure S2.

**Figure 3:**
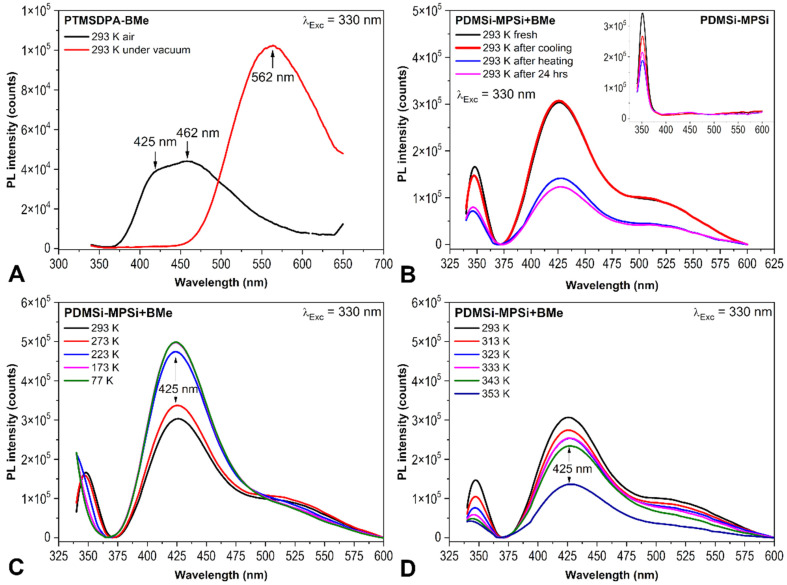
Emission spectra of PTMSDPA+BMe in the air and under vacuum (**A**) and PDMSi-MPSi+BMe (**B**–**D**) exposed to cooling/heating procedure. Comparison of spectra after thermal exposure and after next 24 h (**B**), cooling of the film (**C**) and heating of the film (**D**). Excitation wavelength 330 nm.

Since a decrease in PL intensity of borane cluster in PL spectra is connected with the α-transition of MEH-PPV matrix, other polymers PTMSDPA and PDMSi-MPSi were tested in the same experiment. PTMSDPA has a very high molecular weight (10^6^ PTMSDPA vs. 10^3^ PDMSi-MPSi) and glass transition temperature, which is considered higher than 200 °C [35,36]. PTMSDPA is therefore very rigid and temperature-resistant with limited solubility. Figure 3A shows emission spectra of the thin film PTMSDPA+BMe (thickness 46 ± 3 nm), which were collected at room temperature. BMe emission maximum can be found at 425 nm. The maximum at 462 nm is probably a result of the interaction between BMe and PTMSDPA, because the wavelength shifts to PTMSDPA´s emission maximum at 562 nm. Moreover, photoluminescence of PTMSDPA is quenched in the film PTMSDPA+BMe.

As an essential step of PL measurement within any temperature range, the cryostat chamber must be evacuated and then filled with helium. Figure 3A reflects that the intensity of the BMe emission band underwent an extreme drop right after the vacuum was established in the chamber, and no BMe bands (425 nm and 462 nm) were further detected. The elimination of BMe was checked visually under UV lamp (Supplementary Materials Figure S2) and also microscopically. Before heating, the thin film PTMSDPA+BMe had rougher morphology in comparison with the same film after heating. The changes observed after heating were detected across the entire thin film. Moreover, the image taken after heating indicates that the film kept its overall texture, but it does not carry the features ascribed to BMe. Images from optical microscopy and optical profilometry are given in Figures S3 and S4 in Supplementary Materials. Therefore, the origin of the high mobility of BMe molecules in the polymer matrix does not lie only in the changes connected with transition temperatures but also with macromolecular properties. PTMSDPA has *M_w_* in millions and bulky substituents of phenyl and trimethylsilyl-phenyl groups, as depicted in Figure 1. It has already been reported that PTMSDPA has a very large fractional free volume of 26% [37] when compared with other polymers, e.g., PS 3% [38]. Fractional free volume is defined as a portion of free volume between polymer chains (empty space), expressed as *V*–*V*_0_, where *V* is the volume of the system and *V_0_* is the volume occupied by polymer atoms equal to van der Waals volume multiplied by 1.3, relative to *V* and given in percentage [39]. Bulky substituents of PTMSDPA essentially limit a macromolecular chain arrangement and tight packing, which causes an increase in the fractional free volume. Moreover, the arrangement of PTMSDPA chains is influenced by a spin coating process and kinetics of solvent evaporation from the film. It is expected that extremely long macromolecules do not have enough time to relax and that they are arranged loosely during a thin film drying. The resulting film structure is therefore porous. Even though π-stacking interactions can be engaged for stabilization of PTMSDPA+BMe structure, and thus they can influence PL of the film, they are too weak to persist and lock BMe in the film at low pressure or a high temperature.

On the other hand, PDMSi-MPSi is the σ-conjugated semi-conductive polymer characterized by delocalization of electrons along -Si-Si- chain. Figure 3B depicts the situation after cooling and heating the film PDMSi-MPSi+BMe (approx. 180 nm) and the film without BMe (given in a graph inset). There is no difference between the intensity of the *fresh* film and film *after cooling* in BMe emission maximum. However, the cooling process down to 77 K (Figure 3C) brought an increase in the intensity of both BMe (425 nm) and copolymer spectral band (350 nm) [40]. The intensity of BMe was restored to its initial value after the film reached RT again. Following heating up to 353 K (80 °C) caused a gradual decrease in both spectral bands as given in Figure 3D. The BMe intensity rapid decline breakpoint was determined between 343 K (70 °C) and 353 K (80 °C). *T_α_* of PDMSi-MPSi is 50 °C [41], which represents a contradiction with previous findings for MEH-PPV with a strong influence of α-transition on BMe diffusion. However, PDMSi-MPSi is highly prone to UV degradation that can partially occur during PL measurement (*λ_exc_* = 330 nm irradiated the same area of the film). Photodegradation is connected with structural changes, such as rearrangement of chains, scission, and self-recovery. This degradation process could contribute to BMe´s stability in the matrix and inhibit its diffusion regardless of the *T_α_* of the matrix. Further, the results obtained from the PM-IRRAS experiment of the PDMSi-MPSi film (no photodegradation of a film assumed) described below agree with α-transition changes because borane cluster did not resist temperatures higher than 50 °C and diffused out of the film.

### 2.2. PM-IRRAS Experiments

PL experiments showed that BMe clusters in MEH-PPV, PTMSDPA, and PDMSi-MPSi polymer matrices of thin films suffer from ambient temperature and pressure changes. It is convenient to extend the definition of the free volume and compare the results with the PM-IRRAS experiment that provides a direct relation between vibrational motions of polymers and borane clusters and their response to temperature change. To understand a relation between vibrational motions and free volume, there is a need to define a volume of a polymer system composed of several contributions, as has been summarized in [42]: 1) *V_hc_* is the volume which is occupied by polymer atoms and it is temperature independent. It represents the minimal value of the volume that must be subtracted from the total volume of the system (*V*) to obtain the free volume (*V_free_*). 2) *V_vib_* is vibrational volume and is temperature-dependent and composed of free vibrational volume (*V_free:vib_*) and *V_hc_*. It is a genuine contribution to *V_free_*, as it covers vibrational motions of polymer groups, which occur in both spatially restricted films and various thermodynamic states (glassy, crystalline, …). This contribution is crucial for a reasoning of PM-IRRAS usability. 3) *V_free:exc_* is the excess free volume that represents a total volume without *V_vib_*. It is a volume occupied by a polymer melt.

**Figure 4:**
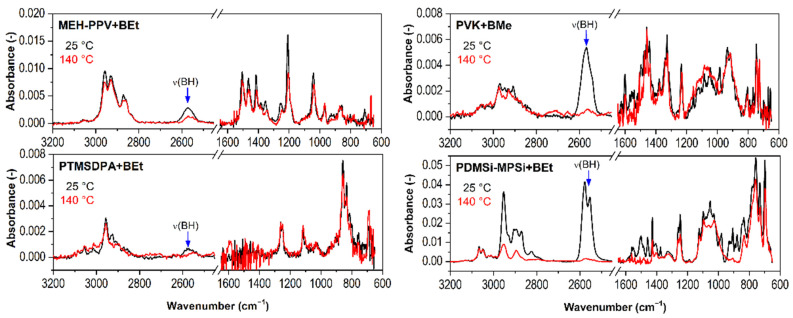
PM-IRRAS spectra of MEH-PPV+BEt, PTMSDPA+BEt, PVK+BMe, and PDMSi-MPSi+BEt at 25 °C and 140 °C (red).

PM-IRRAS spectra of PL polymers with BEt (3,3′,4,4′-Et_4_-*anti*-B_18_H_18_) or BMe measured at 25 °C and 140 °C are given in Figure 4. In addition to MEH-PPV, PTMSDPA, and PDMSi-MPSi, another polymer is involved, PVK, with a high *T_g_* approx. 230 °C [43]. The comparison provides clear evidence that the intensity of B-H stretching (νBH) band at 2660–2500 cm^−1^ significantly decreases with temperature.

**Figure 5:**
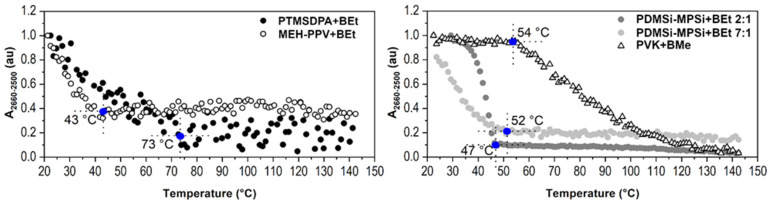
*A_2660-2500_* of B-H stretching in dependence on temperature for thin films of PTMSDPA+BEt, MEH-PPV+BEt, PDMSi-MPSi+BEt, and PVK+BMe. Data on *y*-axis have been normalized 1-0.

**Figure 6:**
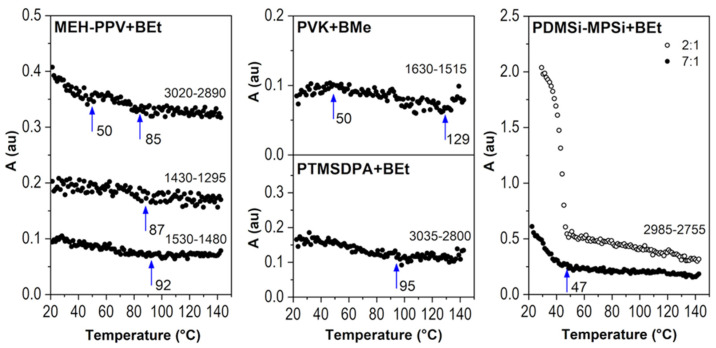
Area values of C-H spectral regions vs. temperature for thin films of MEH-PPV+BEt, PDMSi-MPSi+BEt, PTMSDPA+BEt, and PVK+BMe.

Spectral regions *i* (where *i* is the wavenumber range) were evaluated with the value of their area (*A_i_*). The chosen spectral region was baseline-corrected, and the mathematical area was calculated by integration. The area of the region 2660–2500 cm^−1^ (*A_2660-2500_*) that represents B-H stretching vibrations was plotted against temperature in Figure 5 (the *y*-axis has been normalized for a clear visual comparison). As can be seen, the drop of spectral intensity of B-H stretching band has two different trends. MEH-PPV+BEt, PDMSi-MPSi+BEt, and PTMSDPA-BEt films evince a sharp decrease in *A_2660-2500_* right at the beginning of the measurement at low temperatures. This decrease slows down at 43 °C, 47 °C and 73 °C, respectively. However, only the thin film MEH-PPV+BEt was able to keep a remaining amount of BEt in its structure after heating, as represented by the *A_2660-2500_* value that decreased by 60%. Changes in IR spectra of MEH-PPV+BEt film evoked with heating are mainly connected to C-H stretching and bending vibrations of the polymer at 3020–2980 cm^−1^ and 1430–1295 cm^−1^, respectively, whose response to heating of the film is demonstrated with corresponding area values in Figure 6 (left). Borane cluster diffusion out of the film breaks at 43 °C, which corresponds with a slight change in the trend of *A_3020-2980_* and its break at 50 °C. An abrupt reduction of the amount of borane clusters in the structure of the film adjusts the vicinity of MEH-PPV chains and provides an unoccupied free space between polymer chains and therefore more free volume. Following response in the vibrational free volume is adequate. Moreover, *T_α_* of MEH-PPV is reached and this transition is assigned to another change in a course of both *A_3020-2980_* and *A_1430-1295_* found at 85 °C and 87 °C, respectively. C_ar_-C_ar_ stretching of phenylene-vinylene (demonstrated by *A_1530-1480_* vs. temperature) evinced similar change at 92 °C. Since no transition (α-transition as well) is defined with the only one temperature value, they are usually grasped as temperature ranges or domains with onset, middle, and end temperatures. Temperature values observed by us reflect so. The important structural difference of MEH-PPV among other studied polymers is that the backbone is formed with phenylene rings. An employment of π-stacking interaction between phenyl in backbone and BEt probably helped to attain stability of this thin film at higher temperatures (43 °C–140 °C, *A_2660-2500_* held at 40% of the initial value).

PM-IRRAS spectra of PTMSDPA-BEt at 25 °C and 140 °C do not differ significantly, as shown in Figure 4. These spectra were loaded with a high level of noise, which is also observable in the plot of *A_2660-2500_* vs. temperature (Figure 5), where highly scattered data are present. The high level of data scattering is caused with a setting of a spectral data collection (16 scans and resolution), which provides an efficient speed of scanning, but, unfortunately, can result in an inefficient signal-to-noise ratio. Despite this drawback, a clear decrease in BEt band area is evidenced. The small variance between spectral regions of PTMSDPA polymer suggests a glassy state of side groups, as well as backbone. The course of *A_3035-2800_* (C-H stretching) trend in Figure 6 (middle bottom) thus encourages the assumption of the increase in free vibrational volume with decreasing BEt amount in the film. Therefore, the diffusion of BEt out of the film is presumably connected with a porous structure and significantly higher free volume of this polymer matrix.

The thin film PVK+BMe behaved differently, and the initial value of *A_2660-2500_* held at maximum up to 54 °C, as can be found in Figure 5. Then, a gradual decrease started, resulting in a drop of *A_2660-2500_* to almost 0% of the initial value at 130 °C. This polymer was in glassy state during measurement. However, the sub-*T_g_* structural relaxations of PVK [44] were expected because of a precipitous release of BMe. These adjustments are related to region 1630–1515 cm^−1^, which shows the most substantial changes in IR spectral bands with heating of the film (given in Figure 6, middle-top). Bulky carbazole side group (*A_1630-1515_*), which could serve as a stacking location for BMe clusters, is supposed to move slightly at around 50 °C, which matches perfectly with the decrease in BMe area at 54 °C.

PDMSi-MPSi+BEt was prepared in two different concentrations of polymer:borane cluster; in detail, thin films with a weight ratio 2:1 and 7:1 were prepared. As can be seen in Figure 5, the film with a higher amount of BEt (2:1) keeps the *A_2660-2500_* values stable up to 40 °C, drops within the next 5 °C of heating to 10% of the initial area value, and slowly reaches almost 0% of the initial area value. On the other hand, the film with lower concentration of BEt (7:1) was not able to keep the initial value of *A_2660-2500_* as it gradually went down to 20% of its initial value. However, this decrease stopped at 52 °C and further stabilized. Different trends for two concentration of BEt were observed, which is very probably connected with glass transition domain of copolymer, which was determined 63 °C (see Figure S5 of DSC standard measurement 10 °C/min, done at our laboratory with Labsys Evo DTA/DSC, Setaram). Substantial changes in IR spectra of PDMSi-MPSi were adapted during the heating and are predominantly connected with C-H stretching vibrations at 2985–2755 cm^−1^. As can be seen in Figure 6 (right), they follow the decrease in BEt spectral band.

The diffusion of borane clusters within thin films under heating is, on one hand, not surprising, as low molecular species diffusion and migration within solids has already been observed and described [45,46]. On the other hand, this behavior offers a powerful description of a temperature behavior of not only clusters themselves but also polymer matrices. Therefore, we conducted our experiments with thin films made of polystyrene, which was chosen for being a well-described polymer; factors that influence the borane cluster diffusion were then described.

#### 2.2.1. Type of Borane Cluster

**Figure 7:**
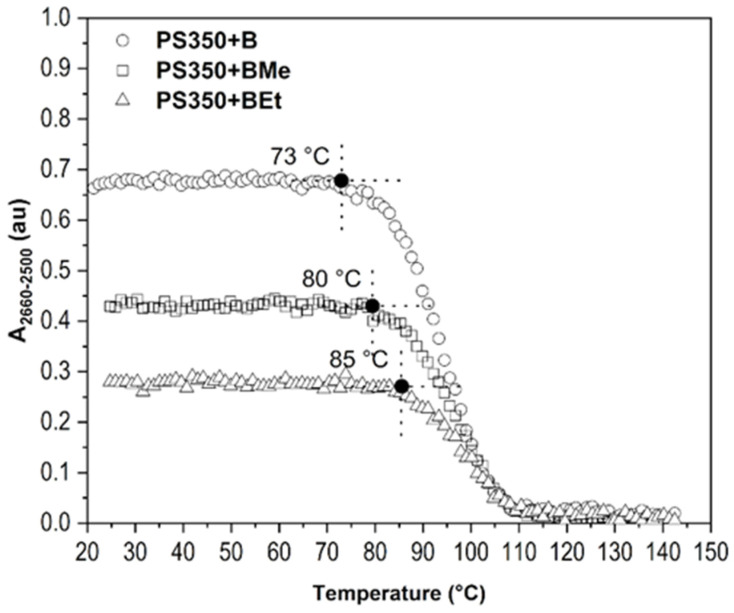
*A_2660-2500_* of B-H stretching in dependence on temperature for thin films PS350+B, PS350+BMe and PS350+BEt. PS350:borane cluster = 10:1. The influence of borane cluster´s hydrogen substitution.

**Figure 8:**
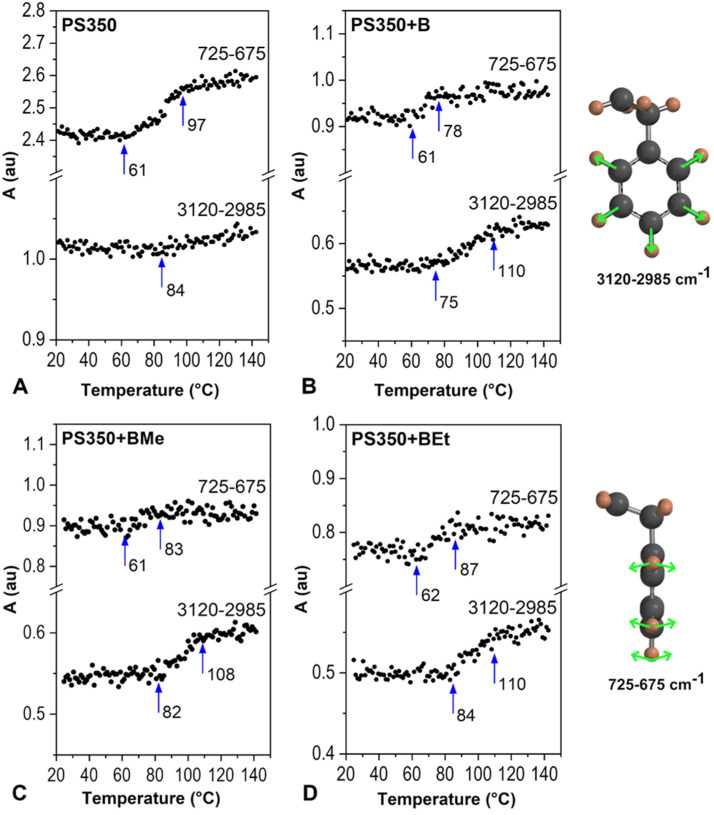
Areas of spectral regions of C_ar_-H stretching (3120–2985 cm^−1^) and out-of-plane bending (725–675 cm^−1^) of PS350 (**A**) and PS350+B (**B**), PS350+BMe (**C**) and PS350+BEt (**D**). PS350:borane cluster = 10:1. Calculated from PM-IRRAS spectra measured in the vacuum.

Borane clusters were studied in the thin film made of PS350 matrix by PM-IRRAS measurement in the vacuum. The same method of a spectral data evaluation, that uses the values of the area of specific regions, was applied. The set of borane molecules, B, BMe, and BEt differs in the hydrogen substitution in the position 3,3´,4,4´- as given in references below. The evaluation of *A_2660-2500_* under increasing temperature is given in Figure 7. It can be stated that PS350 matrix is more convenient for an accommodation of borane clusters of all studied types when compared with results obtained for photoluminescent polymers. PS350 has bulk *T_g_* 108 °C (DSC, standard measurement 10 °C/min, Figure S5) and low free volume, which creates a more suitable environment for small molecules accommodation. However, the heating-induced diffusion cannot be fully avoided. Longer organic substituents improve a compatibility with PS350 matrix and act as compatibilizers between inorganic borane cluster and organic polymer leading to a higher temperature stability. A clear enhancement of the temperature stability of borane cluster in the thin solid film is seen within the row B → BMe → BEt. A sharp decrease in B-H stretching area (*A_2660-2500_*) is detected at 73 °C, 80 °C, and 85 °C, respectively. These temperatures correspond to a temperature domain of β-transition of PS350 as we have described previously [26] and now in Figure 8A. All three films were able to retain borane molecules in the structure up to 110 °C, which, in addition, correlates with *T_g_* (*T_α_*) of PS350.

Areas of regions 3120–2985 cm^−1^ and 725–675 cm^−1^, which are assigned to C_ar_-H stretching and C_ar_-H out-of-plane bending, respectively, manifest a considerable respond to a temperature escalation. The temperature range of the PS350 β-transition domain was determined to be 61 °C–97 °C with maximum change (increase) in free vibrational volume at 84 °C. The first interval, 61 °C–97 °C, was extracted from the response of out-of-plane bending vibration, i.e., *A_725-675_* vs. temperature. This temperature range represents the changes in free vibrational volume caused by a sterically extensive out-of-plane vibration of C_ar_-H of bulky phenyl side groups, which are influenced with non-bonding π-π interactions of the phenyl rings as well. The whole system of phenyl side groups, non-bonding interactions, and vibrational motions is obviously influenced by the temperature increase. The latter temperature, 84 °C, was determined via the increase in *A_3120-2985_* of C_ar_-H in-plane stretching at a certain temperature. As this vibration does not occupy such a large amount of surrounding space, its interaction with phenyl vicinity is therefore limited. This shows that a certain temperature in β-relaxation must be reached before it manifests its effects in a vibrational free volume. Since 84 °C is almost in the middle of the 61 °C–97 °C range, it is considered as the middle temperature of β-transition with the highest rate of change in free vibrational volume. The course of *A* vs. temperature finely agrees with the results obtained from ellipsometry [47] and molecular dynamics models [48].

Further, the migration of borane clusters observed in thin films PS350+B, PS350+BMe, and PS350+BEt affects the upper temperature limit of PS350´s *A_725-675_*, and at the same time, it transforms the trend in temperature response of PS350´s *A_3120-2985_*, as can be seen in Figure 8B–D. The detected changes can be perfectly correlated with the decrease in borane cluster *A_2660-2500_*, as presented in Figure 7. The area of the region of out-of-plane bending follows the original trend found in spectra of the PS350 thin film; however, differences are observed in the values of the end temperature of β-transition, which shifts to 78 °C, 83 °C, and 87 °C for PS350 film with B, BMe, and BEt, respectively. All these temperatures are a few degrees above the temperature at which the main *A_2660-2500_* decrease in particular borane clusters starts. It is assumed that a diffusion of borane clusters among PS350 macromolecules creates locations with a loose arrangement of chains in the polymer structure and correspondingly enables the increase in free vibrational volume. As a result, the end temperature of β-transition is lower. Longer and bulkier substituents of borane clusters, in our experiment ethyl groups, serve as a steric hindrance, which make a cluster movement more difficult, and thus the temperature limit of BEt spectral drop shifts to higher value. Stretching vibration of C_ar_-H is influenced by the presence of borane clusters as well. A breaking point in the plot of *A_3120-2985_* vs. temperature of PS350+B, PS350+BMe, and PS350+BEt shifted to 75 °C, 82 °C, and 84 °C, respectively. If compared with temperatures extracted from Figure 7 (73 °C, 80 °C, and 85 °C), there is again a clear relation. In addition, a new turning point is observed in these plots at 110 °C, at which the B-H stretching band was not detected anymore (*A_2660-2500_* value is close to zero) in PM-IRRAS spectra of all three thin films.

#### 2.2.2. Concentration of Borane Cluster

**Figure 9:**
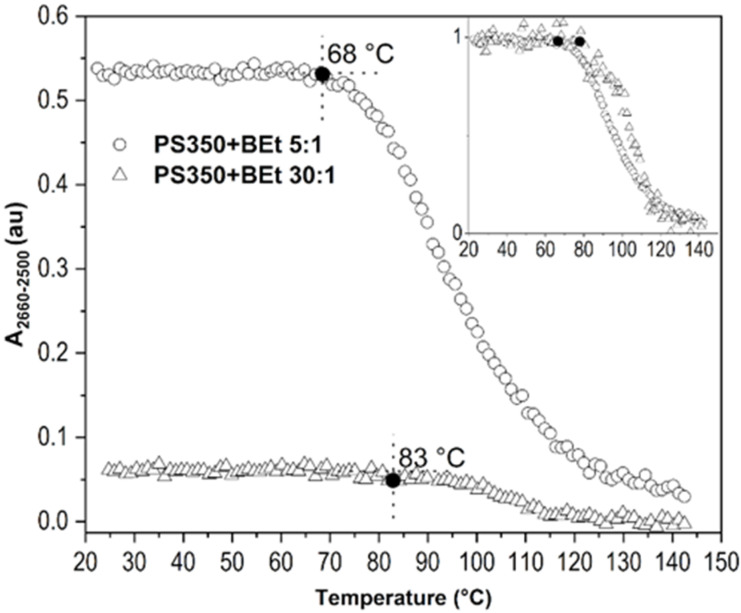
*A_2660-2500_* for B-H stretching as dependent on temperature for thin films of PS350+BEt with PS350:BEt weight ratios of 5:1 (empty triangles) and 30:1 (filled triangles). The influence of borane cluster concentration.

Another experiment indicated the relation between the concentration of BEt in thin film PS350+BEt and its temperature behavior. BEt was chosen for this investigation as the borane cluster that attained the highest temperature stability of all three investigated compounds. Two films with PS350:BEt weight ratios of 5:1 and 30:1 were studied. The plot of *A_2660-2500_* rendered against temperature is depicted in Figure 9. The difference between initial values of *A_2660-2500_* was caused by the different amounts of borane clusters in the film. Film deposition solutions were prepared from the same amount of PS350, but different amounts of BEt. The 5:1 film attained an initial value for *A_2660-2500_* of up to 68 °C, while the 30:1 film kept its stability up to 83 °C. For better comparison of an overall performance of both films, an inset in Figure 9 shows the same data with normalized 1-0 *y*-axis. The 30:1 film evinced higher stability and slower borane cluster diffusion out of the film up to 115 °C. This suggests that a higher portion of BEt within the PS350 matrix does not provide any significant improvement of the BEt stability in the thin film.

#### 2.2.3. Molecular Weight of Polymer

**Figure 10:**
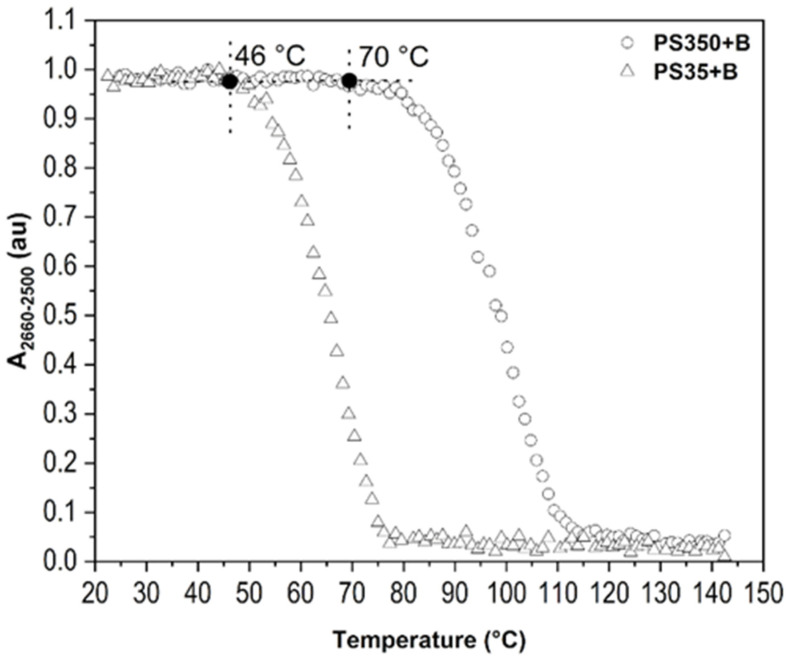
*A_2660-2500_* of B-H stretching as dependent on temperature for thin films of PS350+B (10:1) and PS35+B (10:1). The influence of molecular weight of polymer.

The molecular weights of polymer PS350 (Mw ~ 350,000) and PS35 (Mw ~ 35,000) were another factor of interest. Figure 10 demonstrates that the length of macromolecular chains undoubtedly affects the ability of polymer matrix to accommodate borane clusters. P350+B and PS35+B (both 10:1) kept their initial value of *A_2660-2500_* up to 70 °C and 46 °C, respectively. This proves that the longer chains are able to create more entanglements even in spin-coating and film-drying conditions, which prevents the diffusion of borane clusters up to β-transition of matrix. The assumption that PS chains go through β-transition is supported with results for PS35 thin film, which has lower bulk *T_g_* 61 °C (Figure S5 in Supplementary Materials). The decrease in *A_2660-2500_* at 46 °C thus correlates with lower bulk *T_g_* (*T_α_*). Moreover, borane cluster does not carry any substituents, which could make entanglements easier and diffusion slower. The diffusion is consequently influenced only by factors related with polymer chains.

### 2.3. Improvement of Thin Film Temperature Stability

**Figure 11:**
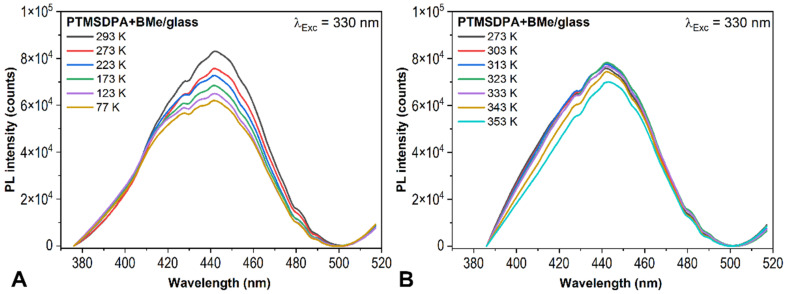
Emission spectra of PTMSDPA+BMe/glass. Excitation wavelength 330 nm. Cooling of the film (**A**), heating of the film (**B**).

The diffusion of borane clusters within thin films is a factor, which considerably limits the viability of the prepared films. Therefore, precautions that lead to reduction of borane clusters mobility within polymer matrix, were tested. Figure 11 depicts the emission spectra during cooling and heating of PTMSDPA+BMe, prepared as written above, with the difference that the thin film was covered with a glass slide. A clear improvement of BMe emission stability (maximum at 425 nm and 443 nm) was achieved. Figure 11B shows a negligible decrease in PL intensity during heating up to 353 K (80 °C). The glass obviously served as a physical barrier for BMe diffusion, which was stopped by this way.

**Figure 12:**
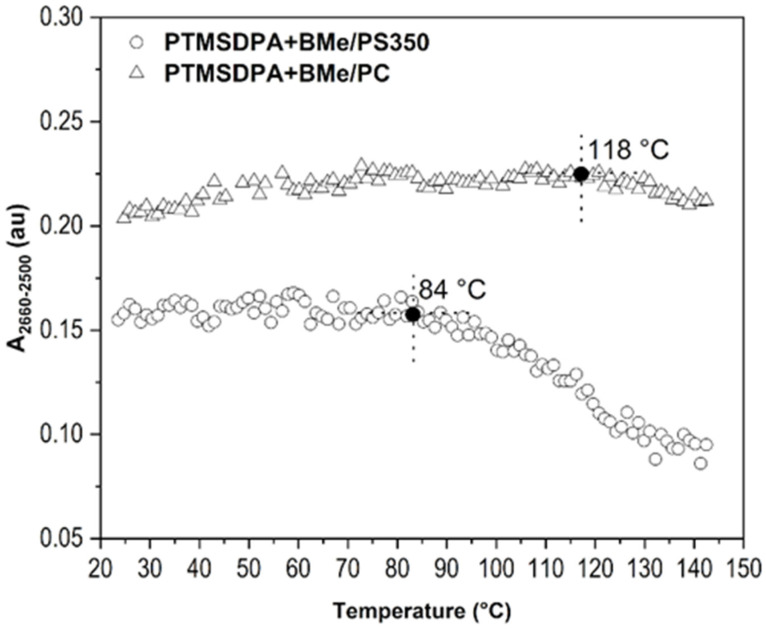
*A_2660-2500_* of B-H stretching as dependent on temperature for thin films of PTMSDPA+BMe/PS350, PTMSDPA+BMe/PC.

Regarding the fact that overlaying the thin film with a glass slide is a coarse solution, even though it might be useable in some applications, we tested another way to improve the temperature resistance of our investigated thin films that is more suitable for electronic applications. This solution lies in a preparation of a two-layered film made of a layer of interest, PTMSDPA+BMe, and a cover layer made of another polymer, in this case PS350 and PC. For dissolution of PS350 and PC, 1-chloropentane and chloroform were used, respectively. As PTMSDPA is poorly soluble in both mentioned solvents (requiring several days), a blending of already-dried PTMSDPA-BMe with the deposited PS350 or PC layer was not expected. Figure 12 shows the result of PM-IRRAS and the dependence of *A_2660-2500_* on the temperature. A slight improvement of BMe detectability (up to 84 °C), and thus its stable embedding in the PTMSDPA matrix can be seen for the film covered with PS350 (*T_g_* = 108 °C), whereas a substantial positive adjustment of BMe stability (up to 118 °C) was attained with PC (*T_g_* = 142 °C, DSC curve in Figure S5) as the cover layer. In respect to the difference between bulk *T_g_* and corresponding sub-*T_g_* relaxations of overlaying polymers, it can be stated that the side-chain localized relaxation of polymers (β-transition) was the main trigger/brake of the borane cluster migration within the thin layers. Borane clusters were detectable in the films until the overlaying polymer underwent β-relaxation, which unlocked the physical barrier for movement of borane cluster molecules. Given this knowledge, one can opt for the appropriate combination of polymers that carry needed PL properties and substantially limit the borane cluster migration at higher temperatures or pressures.

Generalization of this solution, not only for borane clusters, consists in an overlaying (or encapsulation) of the low molecular species in the film; on the other hand, it is necessary to keep their PL properties as well, especially if thin films are supposed to be used in electronics. Even though these requirements can be contradictory, the preparation of active layers for PL devices involves work with electrodes, which could serve as a functional cover layer, working as a migration barrier. This solution is viable if it is possible to deposit the electrode at ambient pressure and room temperature.

## 3. Materials and Methods

### 3.1. Materials

Thin films were prepared from the following polymers and *anti*-octadecaborane(22) and its derivatives. Polystyrene with molecular weight 350 000 g/mol (PS350; *M_w_* ~ 350,000, *M_n_* ~ 170,000) and 35,000 g/mol (PS35; *M_w_* ~ 35,000), poly(N-ethyl-2-vinylcarbazole) (PVK; *M_w_* ~ 990,000), and poly [2-methoxy-5-(2-ethylhexyloxy)-1,4-phenylenevinylene] (MEH-PPV; *M_n_* 40,000–70,000) were purchased from Sigma Aldrich. A high-molecular-weight polymer poly [1-phenyl-2-[p-(trimethylsilyl)diphenyl]acetylene] (PTMSDPA, *M_w_* ~ 10^6^) was prepared at the Faculty of Science of the Charles University in Prague according to the procedure described in [35]. Poly(dimethylsilane-methylphenylsilane) (PDMSi-MPSi; *M_w_* ~ 82,000, *M_n_* ~ 18,000) was obtained from Fluorochem Ltd., and polycarbonate Makrolon (PC, *M_w_* ~ 58,000, *M_n_* ~ 25,000) was obtained from Bayer. Octadecaborane *anti*-B_18_H_22_ (B, *M* = 216.58 g/mol) and its tetra-alkylated derivatives 3,3′,4,4′-Et_4_-*anti*-B_18_H_18_ (BEt, *M* = 328.66 g/mol) and 3,3′,4,4′-Me_4_-*anti*-B_18_H_18_ (BMe; *M* = 272.62 g/mol) were synthesized at the Institute of Inorganic Chemistry of the AS CR [25].

Our 1-chloropentane (99%), toluene (p.a.), and cyclohexane anhydrous (99.5%) stock was purchased from Sigma-Aldrich. Chloroform (stabilized with 2-methyl-2-butene, 99.8%) was obtained from Avantor. All these solvents were used for the preparation of solutions for spin coating.

### 3.2. Thin Film Preparation

Thin films were prepared from solution by spin-coating using a Laurell WS-650-MZ-23NPP instrument. The static method of the solution deposition on the substrate was used, and the spin rate and acceleration were set to 1000 rpm and 500 rpm/min, respectively. Table 1 summarizes data of a preparation of all solutions of polymer with borane clusters (designated as polymer + borane cluster) used for spin-coating. All solutions were prepared and stirred until complete dissolution at laboratory temperature. Toluene and 1-chloropentane were used to prepare PS350 and PS35 solutions with borane clusters. PTMSDPA was dissolved in anhydrous cyclohexane, while MEH-PPV and PVK were dissolved in chloroform to prepare polymer + borane cluster solutions. The choice of solvent was based on the need for an efficient dissolution of polymers and borane clusters and obtaining as smooth a structure of thin films as possible.

Thin films were deposited on quartz glass (QG; 1 cm × 1.5 cm), gold (Au; size 2 cm × 2.5 cm), and silicon (Si; 1 cm × 1 cm) substrates and were further investigated by photoluminescence (PL) spectroscopy, polarization-modulated infrared reflection–absorption spectroscopy (PM-IRRAS), and mechanical profilometry for a determination of a thickness. A cleaning of substrates preceded the spin coating. Substrates were cleaned with Hellmanex (2% of Hellmanex in demi-water), demi-water, acetone (p.a., Avantor, Radnor Township, PA, USA), and isopropanol (p.a., Sigma-Aldrich, St. Louis, MO, USA) in an ultrasonic bath for 10 min/each solvent. The cleaning procedure ended with a 10 min ozone cleaning. After deposition, the films were dried at laboratory temperature in a vacuum oven for approx. 24 h to assure complete solvent evaporation. Solvent evaporation was confirmed with the PM-IRRAS method used for spectroscopic experiments. Infrared spectra of freshly prepared films (after 24 h of solvent evaporation in a vacuum oven) measured at room temperature were compared with spectra collected after the heating of thin films. Besides changes of spectral bands corresponding to B-H stretching of borane clusters (discussed in the paper), no other changes indicating the presence and gradual removal of solvent during heating of the film were observed.

Due to the chemical nature of borane clusters, the whole film preparation procedure was conducted in a glove box under a nitrogen atmosphere because the investigated compounds are highly prone to hydrolysis [49].

### 3.3. Film Thickness and Surface Structure

The thickness of films was measured with a mechanical profilometer Dektak XT-E (Bruker, Billerica, MA, USA) with 1 nm resolution. Thin films were prepared for this purpose on Si substrates to avoid deterioration of the films deposited on Au and QG substrates for spectroscopic measurements. The thickness measurement was set up with a tip radius of 2.5 μm, length of analyzed region 600 µm, and stylus force 3 mg. The thickness was calculated as the average value of the three measurements. The summary of the thickness of particular films is given in Table 1. Except for two films for PL measurement, the thickness is higher than 100 nm. Studies have proved that the thickness of the thin film does influence the glass transition of the polymers; however, the thickness effect is pronounced with layers thinner than 100 nm [10,50]. For this reason, the thickness of studied films is here not considered as a primary factor that would influence the glass transition of the studied polymers.

### 3.4. Photoluminescence Spectroscopy

Photoluminescence (PL) spectra were collected with an FLS920 fluorimeter (Edinburgh Instruments, Edinburgh, Scotland) in helium at various temperatures, provided by the cryostat Optistat DN-V LN2 (Oxford Instruments, Oxford, UK), Oxford Instruments. Thin films on QG substrates were analyzed during cooling down from RT to 77 K (controlled by liquid nitrogen), followed by heating up to 353 K. Emission spectra were collected as follows: (1) immediately after film preparation (labeled *fresh*); (2) during film cooling at particular temperatures; (3) after cooling at RT (labeled *after cooling*); (4) during heating at certain temperatures; (5) after spontaneous cooling down to RT (labeled *after heating*); (6) after 24 h in the cryostat chamber without opening (labeled *after 24 h*).

### 3.5. Polarization-Modulation Infrared Reflection–Absorption Spectroscopy

Infrared spectra of thin films on Au substrates were collected using a Nicolet iN10/iZ10 spectrometer (Thermo Scientific, Waltham, MA, USA) equipped with a heated chamber Refractor-Reactor (Harrick, Pleasantville, NY, USA) with wedged ZnSe windows enabling grazing angle reflection–absorption measurement (incident angle 75°). All films were analyzed in a vacuum at an increasing temperature. The measurement was performed in the mid-infrared region 4000–650 cm^−1^. Spectra were acquired after 16 scans with a resolution of 4 cm^−1^. The wire grid polarizer on KRS-5 substrate was set to the optimal angle of 90°, providing p-polarized radiation. The temperature of a heated stage and the thin film was controlled by the software EZ-ZONE^®^ Configurator (Watlow, St Louis, MO, USA). The vacuum inside the Refractor-Reactor chamber was attained with a vacuum pump and monitored with a digital manometer. The pressure was held at units of Pa during measurement. The temperature profile was set to heating from RT (after stabilization of the vacuum) to 142 °C with a rate of 2.6 °C/min. The chosen heating rate corresponds to the acquisition of one spectrum per 1 °C of temperature increase with a given resolution and number of scans, which diminishes the effect of a sample heating during a particular spectrum measurement.

## 4. Conclusions

PL and PM-IRRAS spectra were used for a description of temperature effects connected with the structure of substrate-supported thin films made of π-conjugated polymers MEH-PPV, PTMSDPA, PVK, σ-conjugated polymer PDMSi-MPSi, and non-luminescent polymer PS with incorporated *anti*-B_18_H_22_ and its tetra-alkylated derivatives.

Emission spectra of borane-cluster-containing thin films revealed that molecules of borane clusters are prone to diffusion in the film at higher temperature or even at low pressure of the ambient atmosphere. The physical interactions (such as π–π stacking), which can be activated between borane clusters and π-conjugated areas, e.g., phenyl rings, are not able to lock clusters within the polymer macromolecules at higher temperatures, especially when polymer matrix goes through *β*- and *α*-transition. After a certain temperature is reached, borane clusters are released from the thin film, which is detectable by a drop of intensity in its emission spectral band. It was found that changes in emission maxima of both polymer matrix (represented with MEH-PPV, PTMSDPA and PDMSi-MPSi) and BMe are connected with polymer structure, its free volume, and thus transition temperatures, including dynamic relaxations at *T_α_* and *T_β_*.

PM-IRRAS investigation was applied to describe the contribution of polymer functional groups to structure-related change in the films during heating to further develop the discussion on this topic. The results were successfully correlated with transition domains and release of borane clusters from the films prepared of either PL polymers or PS. The following points summarize the results: (i) borane cluster diffusion out of the film is connected with an increase in free vibrational volume (within both β- and α-transition) as proved on PL polymers; (ii) more bulky substituents (ethyl-) attached to borane cluster support its stability in the PS350 matrix up to higher temperature because they can also improve the compatibility of polymer–borane cluster system; (iii) the concentration of borane clusters in the thin film seems not to be a determining factor that would significantly increase the film temperature stability; (iv) higher molecular weight of the polymer promotes the borane cluster stability and limits its migration up to higher temperatures (a difference of 25 °C was found between PS35 and PS350).

Regarding the importance of doped PL polymer layers in the development of electronic devices, a simple but efficient solution was proposed that retards the borane cluster migration out of the films. The thin film with incorporated borane clusters was overlaid with another thin layer of polymer, which served as a physical barrier for the cluster migration. We reached an improvement of temperature stability of borane clusters in the polymer film of 45 °C (PTMSDPA+BEt–73 °C vs. PTMSDPA+BMe/PC–118 °C) and showed that properties of the overlaying polymer, such as β-relaxation, are crucial for triggering or stopping the cluster migration out of the film. This knowledge can be used for a proper designing of devices and their active layers with electrodes that could serve as another type of cover layer.

## Figures and Tables

**Table 1:** Composition of solutions for spin-coating and thin films with film thickness.

Thin Film	Solvent	Solution for Spin Coating		Solid Thin Film	Film Thickness (nm)
		Polymer Weight	Borane Weight	Polymer:Borane Weight Ratio	
		per 1 mL of Solvent			
MEH-PPV+BMe^PL^	chloroform	3.0 mg	1.5 mg	2:1	60 ± 1
PTMSDPA+BMe^PL^	cyclohexane	2.0 mg	1.0 mg	2:1	46 ± 3
PDMSi-MPSi+BMe^PL^	chloroform	20.0 mg	10.0 mg	2:1	180 ± 30
PTMSDPA+Bet	cyclohexane	1.0 mg	0.6 mg	2:1	113 ± 5
MEH-PPV+Bet	chloroform	8.0 mg	4.5 mg	2:1	116 ± 5
PDMSi-MPSi+Bet	chloroform	23.0 mg	10.0 mg	2:1	200 ± 40
PDMSi-MPSi+Bet	chloroform	23.0 mg	3.3 mg	7:1	200 ± 20
PVK+BMe	chloroform	10.0 mg	5.0 mg	2:1	110 ± 5
PS350+B	1-chlorpentane	12.5 mg	1.3 mg	10:1	130 ± 5
	toluene	19.0 mg	2 mg	10:1	190 ± 40
PS35+B	toluene	19.0 mg	2 mg	10:1	140 ± 20
PS350+BMe	1-chlorpentane	12.5 mg	1.3 mg	10:1	120 ± 5
PS350+BEt	1-chlorpentane	12.5 mg	1.3 mg	10:1	100 ± 5
	toluene	30.0 mg	6.0 mg	5:1	358 ± 7
		30.0 mg	1.0 mg	30:1	308 ± 4

PL: sample used for PL spectra measurement.

## Data Availability

The data will be available from the corresponding author following reasonable request.

## Supplementary Materials

The following supporting information can be downloaded at: www.mdpi.com/article/10.3390/ijms23158832/s1.
