# Supplementary material for: Incorporation of the New anti-Octadecaborane Laser Dyes into Thin Polymer Films: A Temperature-Dependent Photoluminescence and Infrared Spectroscopy Study
Source: Int J Mol Sci. 2022 Aug 8;23(15):8832. doi: 10.3390/ijms23158832 (PMC9368784; doi:10.3390/ijms23158832)
Supplement: Supplementary file 1 [file ijms-23-08832-s001.zip › ijms-1815003-supplementary.pdf]

Incorporation of the new *anti*-octadecaborane laser dyes into thin polymer films: A temperature-dependent photoluminescence and infrared spectroscopy study

Tereza Capkova, Barbora Hanulikova*, Jakub Sevcik, Pavel Urbanek, Jan Antos, Michal Urbanek, Ivo Kuritka

Centre of Polymer Systems, Tomas Bata University in Zlin, tr. Tomase Bati 5678, 760 01 Zlín, Czech Republic
* Corresponding author: Barbora Hanulikova, Tel.: +420576031743, Email: hanulikova@utb.cz

Supplementary Materials

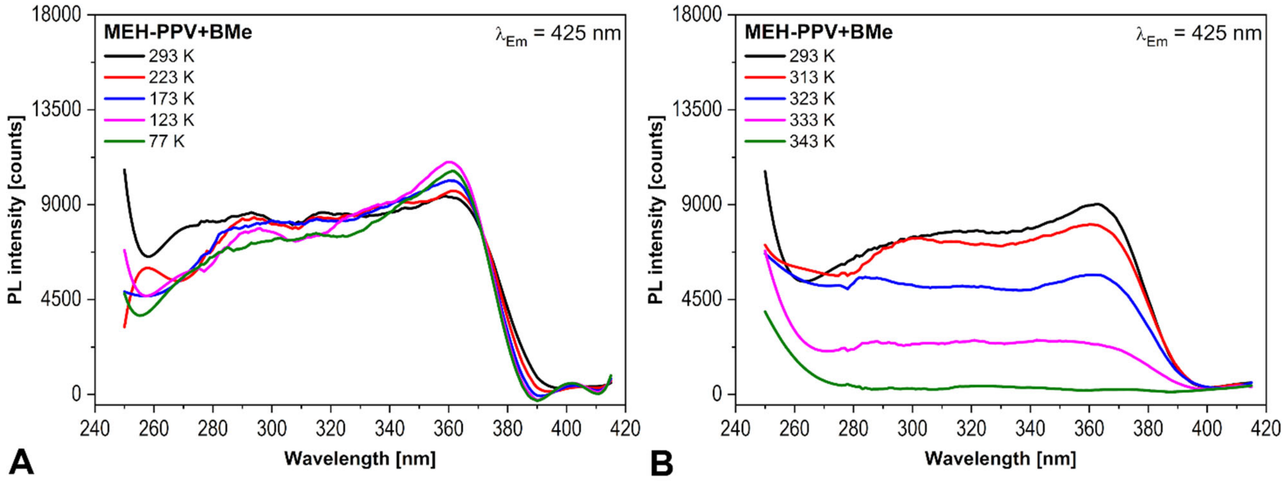

Figure S1: Excitation spectra of MEH-PPV+BMe thin film. Emission wavelength 425 nm. Cooling of the film (A), heating of the film (B).

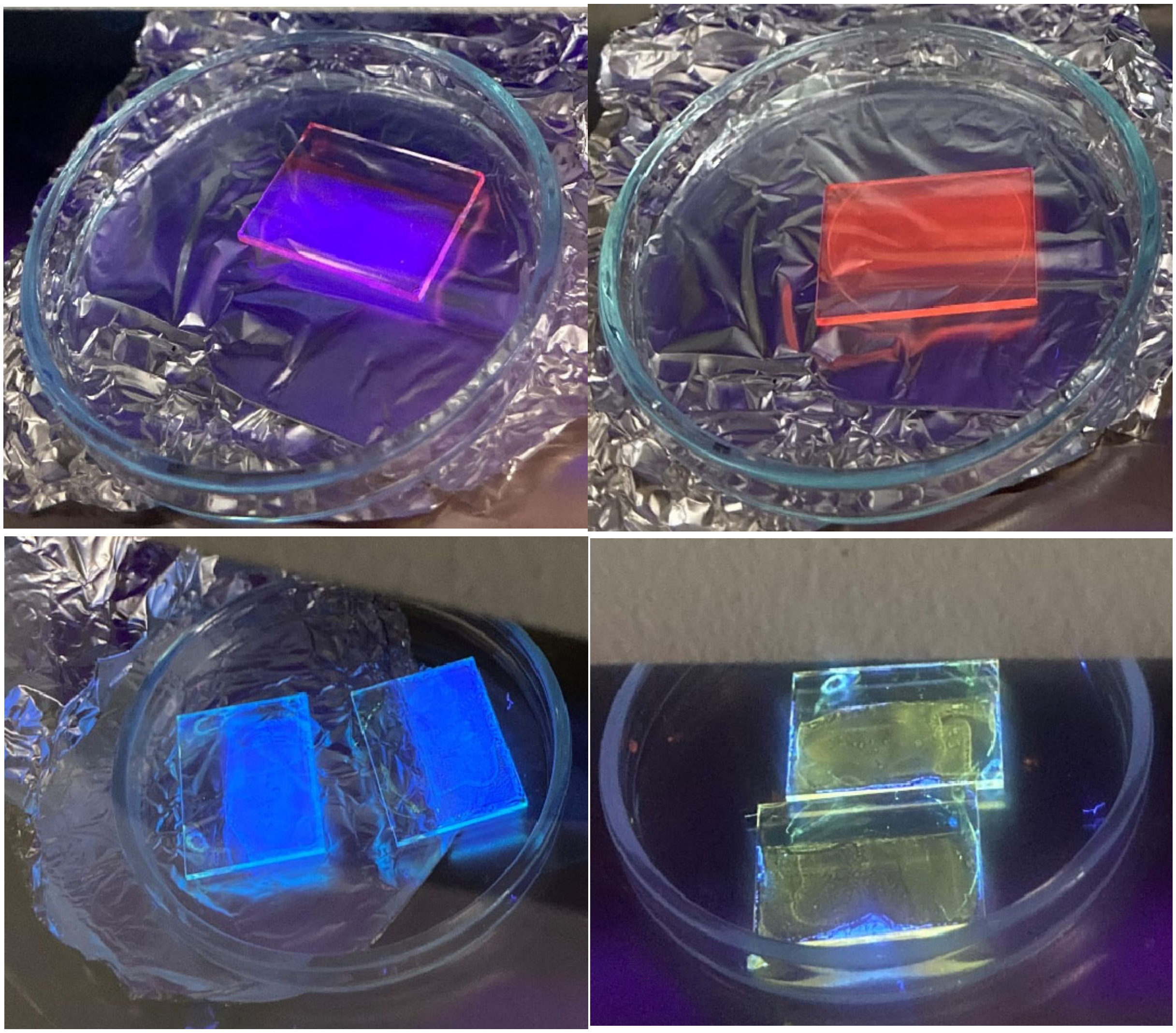

Figure S2: Photographs of MEH-PPV+BMe (top) and PTMSDPA+BMe (bottom) thin films under a UV lamp before heating (on the left) and after heating (on the right) with and without the blue emission of the borane cluster, respectively.

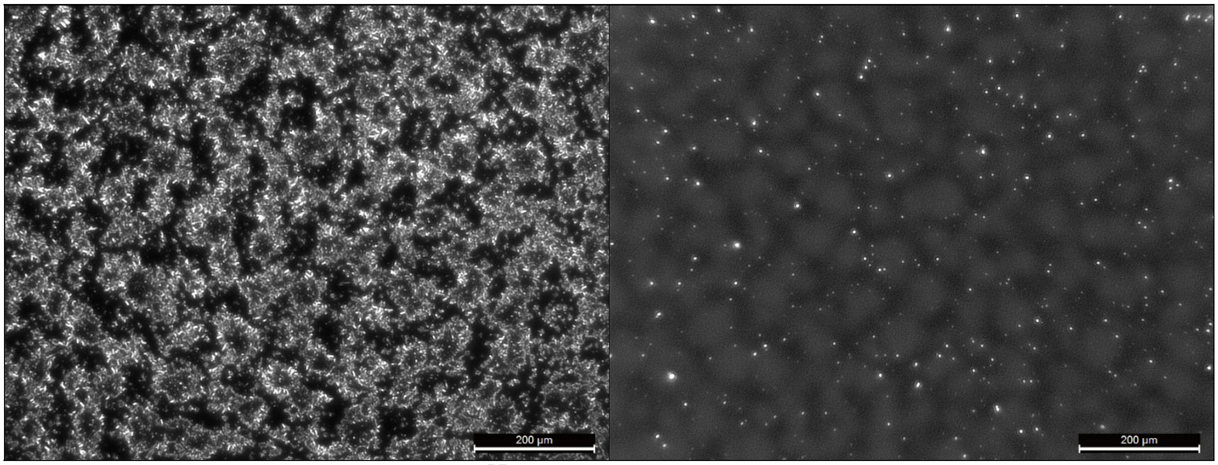

Figure S3: Images of PTMSDPA+BMe^{PL} thin films before (left) and after heating (right). Images were taken with the optical microscope Leica DVM2500 Digital Camera (Leica).

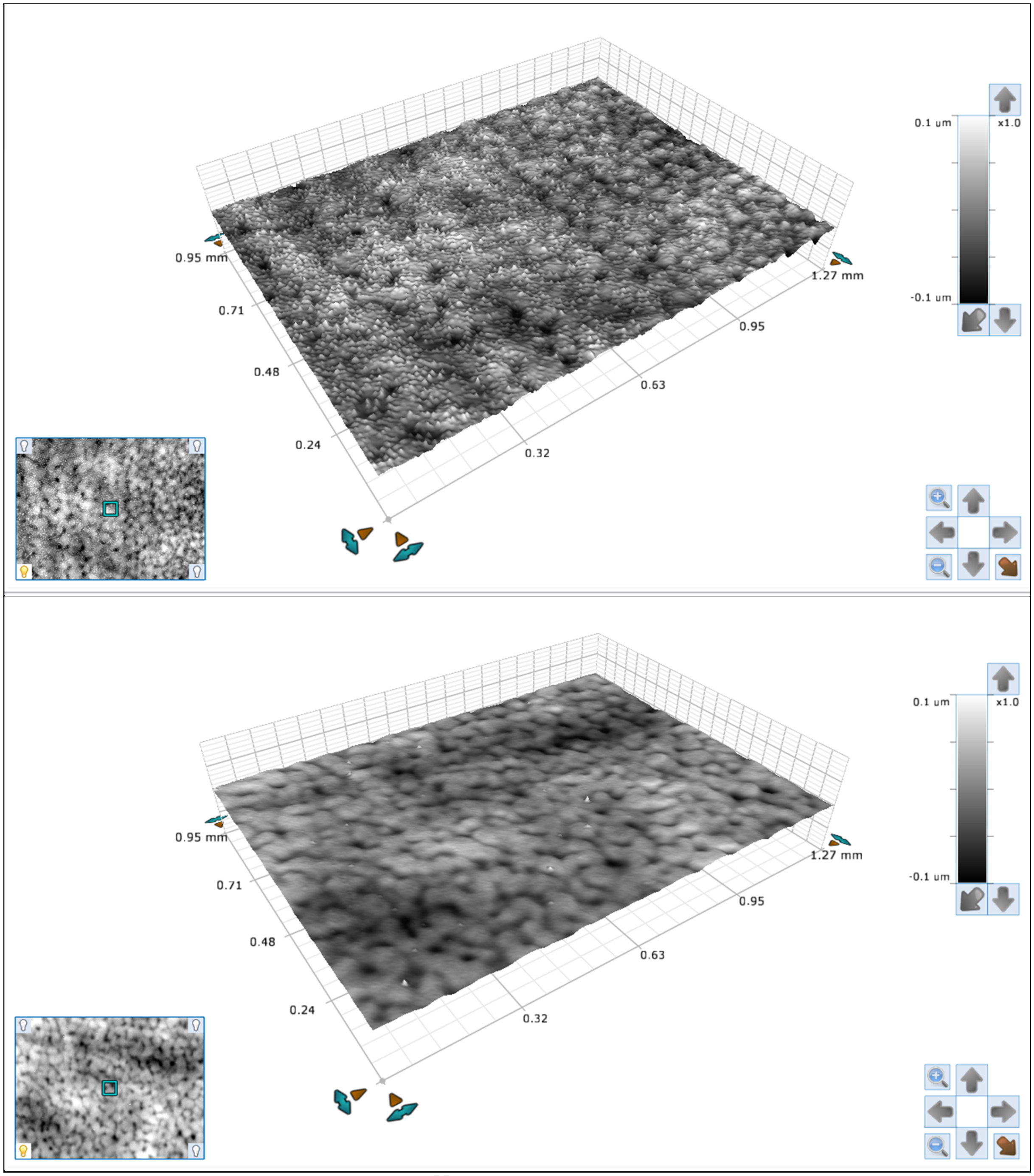

Figure S4: Images of PTMSDPA+BMe^{PL} thin films before (top) and after heating (bottom). Images were taken with the optical profilometer CONTOUR GTK-A (Bruker).

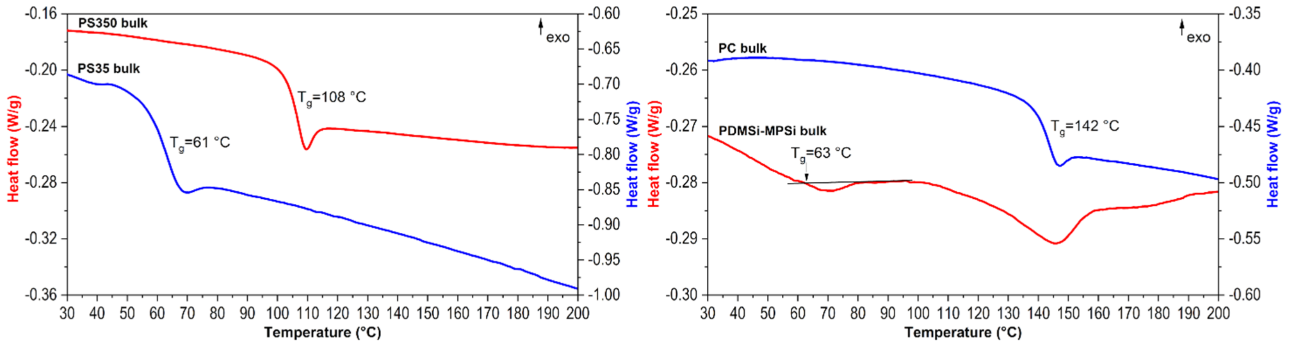

Figure S5: DSC curves of PS350 and PS35 (on the left) and PC and PDMSi-MPSi (on the right). T_g extracted from 2nd heating, heating rate 10 °C/min. DSC curves were measured using LabSys Evo DTA/DSC (Setaram).
